# Thermosensitive Porcine Myocardial Extracellular Matrix Hydrogel Coupled with Proanthocyanidins for Cardiac Tissue Engineering

**DOI:** 10.3390/gels11010053

**Published:** 2025-01-09

**Authors:** José Luis Hidalgo-Vicelis, Angélica Raquel Rivera-Contreras, Beatriz Hernández-Téllez, Gabriela Piñón-Zárate, Katia Jarquín-Yáñez, Tatiana Fiordelisio-Coll, José Manuel Saniger-Blesa, Gertrudis Hortensia González-Gómez, María Alicia Falcón-Neri, María Margarita Canales-Martínez, Andrés Eliú Castell-Rodríguez

**Affiliations:** 1 Laboratory of Immunotherapy and Tissue Engineering, Department of Cellular and Tissue Biology, Faculty of Medicine, National Autonomous University of Mexico, Av. Universidad 3000, Copilco Universidad, Coyoacán, Ciudad de México 04510, Mexico; joseluis.hidalgov@comunidad.unam.mx (J.L.H.-V.); angie_rrivera@ciencias.unam.mx (A.R.R.-C.); bhernandezt12@gmail.com (B.H.-T.); gabrielapinon@unam.mx (G.P.-Z.); katys12@hotmail.com (K.J.-Y.); 2 Laboratory of Comparative Neuroendocrinology, Department of Biology, Faculty of Sciences, National Autonomous University of Mexico, Av. Universidad 3000, Copilco Universidad, Coyoacán, Ciudad de México 04510, Mexico; tfiorde@ciencias.unam.mx; 3 Group of Nanostructured Supports, Department of Micro and Nanotechnologies, Institute of Applied Sciences and Technology, National Autonomous University of Mexico, Av. Universidad 3000, Copilco Universidad, Coyoacán, Ciudad de México 04510, Mexico; jose.saniger@icat.unam.mx; 4 Laboratory of Functional Biophysics, Department of Physics, Faculty of Sciences, National Autonomous University of Mexico, Av. Universidad 3000, Copilco Universidad, Coyoacán, Ciudad de México 04510, Mexico; hortecgg@ciencias.unam.mx (G.H.G.-G.); maryaliciafalcon@ciencias.unam.mx (M.A.F.-N.); 5 Laboratory of Pharmacognosy, Unit of Biotechnology and Prototypes, Faculty of Higher Studies Iztacala, National Autonomous University of Mexico, Avenida de los Barrios 1, Los Reyes Iztacala, Tlalnepantla de Baz 54090, Estado de México, Mexico; dra.margaritacanales@gmail.com

**Keywords:** hydrogel, extracellular matrix, grape seed proanthocyanidins, cardiac tissue engineering

## Abstract

Currently, there are no therapies that prevent the negative myocardial remodeling process that occurs after a heart attack. Injectable hydrogels are a treatment option because they may replace the damaged extracellular matrix and, in addition, can be administered minimally invasively. Reactive oxygen species generated by ischemia-reperfusion damage can limit the therapeutic efficacy of injectable hydrogels. In order to overcome this limitation, grape seed proanthocyanidins were incorporated as antioxidant compounds into a thermosensitive myocardial extracellular matrix hydrogel in this study. For the fabrication of the hydrogel, the extracellular matrix obtained by decellularization of porcine myocardium was solubilized through enzymatic digestion, and the proanthocyanidins were incorporated. After exposing this extracellular matrix solution to 37 °C, it self-assembled into a hydrogel with a porous structure. According to the physicochemical and biological evaluation, the coupling of proanthocyanidins in the hydrogel has a positive effect on the antioxidant capacity, gelation kinetics, in vitro degradation, and cardiomyocyte viability, indicating that the hydrogel coupled with this type of antioxidants represents a promising alternative for potential application in post-infarction myocardial regeneration. Furthermore, this study proposes the best concentrations of proanthocyanidins that resulted in the hydrogels for future studies in cardiac tissue engineering.

## 1. Introduction

One of the consequences of ischemic heart disease is myocardial infarction (MI), which can lead to heart failure and death. According to the World Health Organization, ischemic heart disease is the leading cause of death worldwide [1]. MI is defined as an ischemic event resulting in myocardial necrosis due to partial or total occlusion of the coronary arteries [2]. Decreased or interrupted blood flow to a portion of the heart is usually due to coronary atherosclerosis and thrombosis, leading to myocardial damage [3]. Myocardium damaged during MI cannot regenerate, leading to negative tissue remodeling, including cardiomyocyte death, extracellular matrix (ECM) degradation, fibrosis, ventricular dilation, and thinning of the heart wall, leading to decreased cardiac function [2,4].

Conventional treatments used after an MI are pharmacological therapies (e.g., diuretics, angiotensin-converting enzyme inhibitors, and beta-blockers) that initially help to treat the symptoms of developing heart failure. However, late-stage patients require invasive surgical procedures, such as implantation of ventricular assist devices or heart transplantation. The latter is limited by the shortage of donor organs, immunological rejection, and prolonged hospitalization time [2,5]. On the other hand, through tissue engineering, two clinical approaches have been used for post-MI therapy: cell therapy and cardiac patches. In cell therapy, cells are suspended in saline or culture media and administered in a minimally invasive manner through a catheter into the heart wall. However, tissue necrosis impedes cell adhesion, resulting in decreased cell retention and survival in the infarct region. On the other hand, cardiac patches provide structural support in the damaged ventricle and induce cellular recruitment to the material; however, their implantation is limited to the epicardial surface of the heart and involves an open-chest surgical procedure [6]. Thus, injectable hydrogels are an attractive therapeutic option for cardiac repair, as they can replace the damaged ECM and, in addition, can be administered through a transendocardial injection catheter inserted through the radial or femoral artery. Once it reaches the ventricular chamber, the hydrogel is placed through multiple injections with a needle to distribute it throughout the infarct and the border zone [6,7,8]. And once the hydrogel is injected into the cardiac wall, it must self-assemble in situ at a physiological temperature [2]. This minimally invasive approach to cardiac repair has numerous benefits, including decreased surgical risk, surgery time, local tissue trauma, and recovery time [9].

Biomaterials that have been studied as injectable hydrogels for cardiac tissue engineering include natural materials such as collagen, fibrin, hyaluronic acid, alginate, and chitosan and synthetic materials such as poly(ethylene glycol), polyvinyl alcohol, and poly(N-isopropylacrylamide) [8,9]. Furthermore, these biomaterials have been investigated and combined to improve their physicochemical and biological properties [6]. However, the drawback of these materials is that they do not fully mimic the biochemical composition of the myocardial ECM, which is essential since the ECM not only provides structural support to cells but can also induce signals to guide cell adhesion, proliferation, and maturation [9,10]. For this reason, the deployment of decellularized tissues is an increasingly used strategy in tissue engineering since the ECM obtained from them offers the advantage of being biomimetic, meaning that it mimics the chemical and biological signals of the native microenvironment [4,5,6].

As mentioned, MI occurs when the blood supply to the heart is interrupted. Once blood flow is restored, oxygen in the blood leads to generating reactive oxygen species, leading to cellular damage. Oxidative stress caused by ischemia-reperfusion limits the therapeutic efficacy of cellular hydrogel-based approaches [11]. To overcome oxidative stress, grape seed proanthocyanidins (GSPs) were incorporated into a thermosensitive hydrogel in this study. Among the main biological effects of this type of polyphenolic flavonoids are their antioxidant, anti-inflammatory, anti-calcium, and antimicrobial properties [12]. Currently, some decellularized cardiovascular devices of porcine origin have been coupled with GSPs, such as cardiac valves [13], aortic segments [14], and cardiac patches [15]. However, GSPs coupled into thermosensitive myocardial ECM hydrogels for cardiac tissue engineering have not been studied. Thus, this study aimed to analyze the physicochemical properties of a thermosensitive myocardial ECM hydrogel coupled with GSPs and to evaluate the viability of chicken embryo cardiomyocytes cultured on this three-dimensional scaffold.

## 2. Results and Discussion

### 2.1. Porcine Myocardial ECM

**Figure 1:**
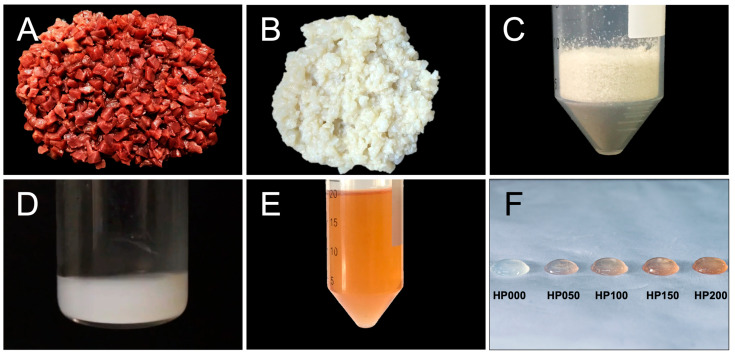
Obtaining porcine myocardial ECM and fabrication of hydrogels coupled with GSPs. (**A**) Ventricular tissue isolated from a porcine heart and cut into small pieces; (**B**) ECM after continuous agitation of the tissue in sodium dodecyl sulfate solution; (**C**) lyophilized and ground myocardial ECM; (**D**) pepsin-digested myocardial ECM powder; (**E**) grape seed proanthocyanidins solution; (**F**) myocardial ECM hydrogels coupled at different GSPs concentrations (HP000: 0 μg·mL^−1^, HP050: 50 μg·mL^−1^, HP100: 100 μg·mL^−1^, HP150: 150 μg·mL^−1^ and HP200: 200 μg·mL^−1^) after incubation at 37 °C.

For hydrogel fabrication, porcine ventricular tissue ECM was obtained by decellularization (Figure 1A,B). The ECM was then lyophilized and pulverized (Figure 1C) and solubilized through enzymatic digestion (Figure 1D), and GSPs were incorporated (Figure 1E). When this ECM solution was exposed to 37 °C, it self-assembled into a hydrogel (Figure 1F).

**Figure 2:**
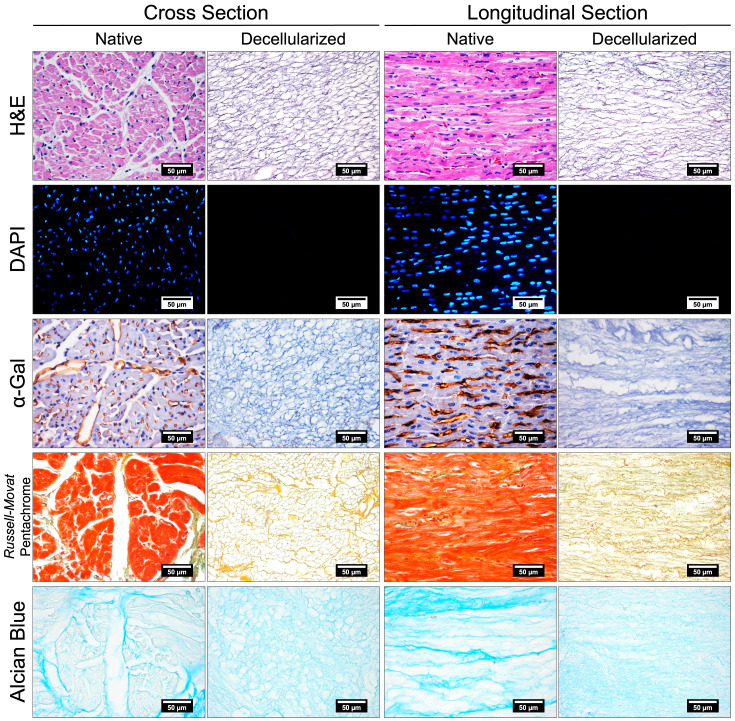
Histologic evaluation of native and decellularized tissue in transverse and longitudinal sections. H&E stain: The cytoplasm and ECM are pink, and the nucleus is violet. DAPI stain: the nucleus is bright blue. Immunohistochemistry to demonstrate α-Gal antigen is brown. Russell-Movat pentachrome stain: nuclei are black, cytoplasm is red, collagen is yellow, elastic fibers are purple to black, and mucopolysaccharides are blue-green. Alcian blue stain: proteoglycans are light blue.

**Figure 3:**
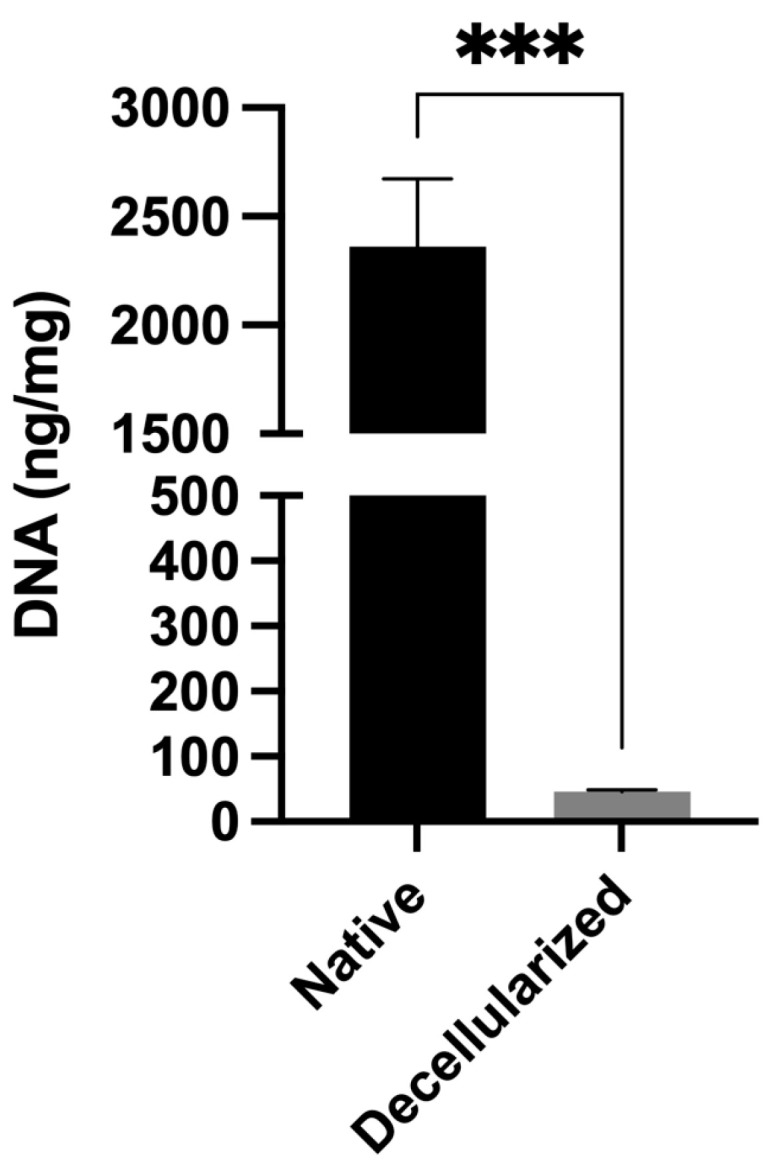
DNA content of native and decellularized tissues. The DNA content of the decellularized tissue was very low compared to the native tissue, with a significant difference between them. In addition, the decellularized tissue had a DNA content below the recommended limit (50 ng·mg^−1^). *** *p* < 0.0002 indicates a significant difference between the treatments, *n* = 3.

Decellularization of xenogeneic tissues, such as the porcine ventricular tissue, is essential for graft acceptance and inducing proper remodeling [16]. Thus, the absence of cells in the decellularized tissue was verified by histology, and the DNA concentration was assessed. H&E and DAPI staining confirmed the absence of cells after subjecting the tissue to the decellularization process (Figure 2). Likewise, the absence of the α-Gal epitope, which is involved in the immunological rejection of xenografts, was verified [17,18]. In immunohistochemistry for α-Gal, we observed that decellularization completely eliminated this molecule located in the endothelium of blood vessels. To quantitatively characterize the degree of decellularization, DNA from native and decellularized tissues was isolated and quantified using the phenol/chloroform/isoamyl alcohol method and a NanoDrop™ 2000 spectrophotometer (Thermo Fisher Scientific Inc., Wilmington, DE, USA), respectively (Figure 3). Native tissue had 2360.8 ± 311.3 ng of DNA per mg of dry tissue, and decellularized tissue had 45.7 ± 3.5 ng of DNA per mg of dry tissue, the difference between them being significant (*** *p* < 0.0002). It is important to note that the DNA content of decellularized tissue was below the recommended limit of 50 ng of DNA per mg of dry tissue for decellularized tissues [19]. Thus, our results showed that the tissue was effectively decellularized, which will minimize potentially negative immunological responses once implanted. Once the decellularization process was verified as effective, we found that the resulting ECM retained some components such as collagen and proteoglycans according to Russell-Movat pentachrome and Alcian blue stains (Figure 2). Some studies have shown that collagen types I and VI are found in greater proportion in the myocardial ECM, while collagen types III, IV, and V are in lesser amounts [20]. On the other hand, the proteoglycans retained in the myocardial ECM are important since, in addition to keeping the ECM hydrated, they also retain growth factors that can stimulate cell viability, proliferation, and differentiation [21].

### 2.2. Physicochemical Evaluation of Hydrogels

#### 2.2.1. Infrared Spectroscopy

**Figure 4:**
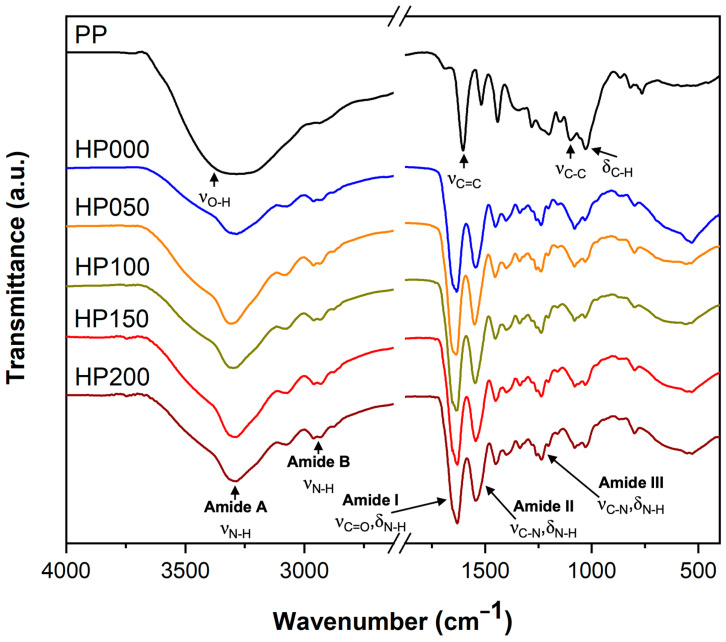
FTIR spectra of GSPs powder (PP) and myocardial ECM hydrogels coupled at different GSPs concentrations (HP000: 0 μg·mL^−1^, HP050: 50 μg·mL^−1^, HP100: 100 μg·mL^−1^, HP150: 150 μg·mL^−1^ and HP200: 200 μg·mL^−1^).

The chemical functional groups present in myocardial ECM and GSPs were identified by Fourier transform infrared spectroscopy (FTIR), and the type of interaction between these components in the hydrogels was confirmed. GSPs (Figure 4, PP spectrum) have bands at 3270, 1629, 1149, and 1030 cm^−1^, indicating stretching of O-H groups, stretching of C=C groups, stretching of C-C groups, and bending of C-H groups, respectively. In the ECM hydrogel spectrum without GSPs (Figure 4, HP000 spectrum), five absorption bands characteristic of collagen, which is the main component of ECM, can be observed [22]. The amide bands A (3285 cm^−1^) and B (2935 cm^−1^) are associated with the stretching vibrations of N-H groups. The amide band I (1650 cm^−1^) is attributed to the stretching vibrations of C=O groups coupled to N-H bending vibrations. The amide II (1545 cm^−1^) arises from N-H bending vibrations coupled to C-N stretching vibrations, while the amide III (1237 cm^−1^) is assigned to both C-N stretching and N-H bending vibrations [23]. The positions of these amide bands of the ECM hydrogels (Figure 4, spectra HP000, HP050, HP100, HP150, and HP200) did not change with increasing GSPs concentration, but the amide A bands were intensified. The above can be attributed to hydrogen bonds formed mainly between the amide and carbonyl groups of collagen and the hydroxyl groups of GSPs [24].

The ratio of absorption intensities of the Amide III band (1237 cm^−1^) and the band at 1450 cm^−1^, shown as A_III_/A_1450_, is considered a measure of preserving the integrity of the collagen triple helices [25]. Table 1 shows that the A_III_/A_1450_ ratios for the GSPs-coupled myocardial ECM hydrogels increase slightly from ≈1.02 for the hydrogel without GSPs to ≈1.07 at most. This suggests that GSPs do not significantly alter the triple helix conformation of collagen, as the ratio for denatured collagen (gelatin) is ≈0.6 [24]. According to previous studies, collagen–water hydrogen bonds are crucial for stabilizing the triple helix structure of collagen [26]. Therefore, GSPs (rich in hydroxyl groups) could displace water to create new hydrogen bonding interactions with collagen, thus preserving its helical structure.

#### 2.2.2. Antioxidant Capacity

**Figure 5:**
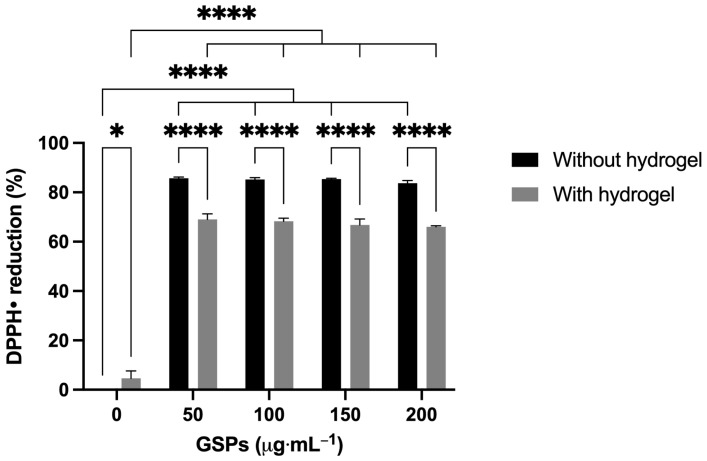
Percentage of DPPH• reduction at different GSPs concentrations coupled or not to the myocardial ECM hydrogel. * *p* < 0.0424 and **** *p* < 0.0001 indicate a significant difference between the treatments, *n* = 3.

The antioxidant capacity of myocardial ECM hydrogels was determined by the 2,2-diphenyl-1-picrylhydrazyl (DPPH•) assay. The DPPH• assay is a colorimetric method that evaluates the antioxidant capacity of a substance by its ability to scavenge DPPH• free radicals [27]. When antioxidants react with DPPH•, it is reduced to (DPPH-H), and the solution is discolored from purple to yellow [28]. The higher the discoloration is, the higher is the reducing capacity of the antioxidant. First, the antioxidant capacity of the GSPs was determined. Figure 5 shows that the GSPs not coupled to the hydrogel showed a high percentage of DPPH• reduction (85.0 ± 0.9%), and the percentage was similar among the different concentrations. Given the observed trend, the concentration at which DPPH• was reduced by 50% (IC_50_) and the antioxidant activity index (AAI) of the GSPs were experimentally determined, whose values were 15.0 μg·mL^−1^ and 2.0, respectively, indicating that GSPs have a strong antioxidant activity [29] and that a low concentration (>15.0 μg·mL^−1^) was sufficient to obtain a high percentage of DPPH• reduction. Likewise, Figure 5 shows that the GSPs coupled to the hydrogel also exhibited a high percentage of DPPH• reduction (67.5 ± 2.3%). However, the percentage was lower than the GSPs not coupled to the hydrogel. The decrease in the percentage of DPPH• reduction by the GSPs coupled to the hydrogel can be attributed to the fact that a part of the hydroxyl groups of the GSPs, which are responsible for the antioxidant activity, are interacting with the amide and carbonyl groups of the collagen through hydrogen bonds [24]. On the other hand, the hydrogel not coupled with GSPs (0 μg·mL^−1^) presented a very low percentage of DPPH• reduction (4.6 ± 3.0%), possibly because the myocardial ECM exhibits intrinsic antioxidant activity [30]. The fact that GSPs-coupled hydrogels have shown antioxidant capacity implies two things: (1) that the hydrogels will be more stable against ROS caused by ischemia-reperfusion and will be able to better perform their function as cellular scaffolds, and (2) the same hydrogel will mitigate oxidation in the myocardium and protect cells from the harmful effects of ROS.

#### 2.2.3. Scanning Electron Microscopy

**Figure 6:**
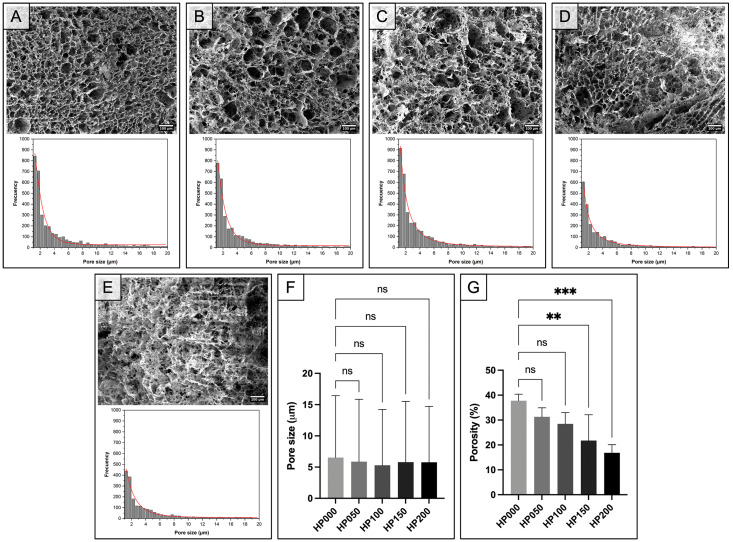
Morphology and pore size analysis of myocardial ECM hydrogels coupled with GSPs. Representative SEM micrographs of the cross-section and histograms of the pore size distribution of hydrogels coupled at different GSPs concentrations (*n* = 4): (**A**) 0 μg·mL^−1^; (**B**) 50 μg·mL^−1^; (**C**) 100 μg·mL^−1^; (**D**) 150 μg·mL^−1^; (**E**) 200 μg·mL^−1^. The histograms show that the number of pores decreases with increasing GSPs concentration due to cross-linking interactions. (**F**) Pore size and (**G**) porosity percentage of hydrogels coupled at different GSPs concentrations (HP000: 0 μg·mL^−1^, HP050: 50 μg·mL^−1^, HP100: 100 μg·mL^−1^, HP150: 150 μg·mL^−1^ and HP200: 200 μg·mL^−1^), *n* = 4. ns indicates no significant difference between the treatments. ** *p* < 0.0084 and *** *p* < 0.0008 indicate a significant difference between the treatments.

The effect of GSPs on the morphology of myocardial ECM hydrogels was observed by scanning electron microscopy (SEM). SEM observation showed that hydrogels coupled at different GSPs concentrations self-assembled into a porous structure with various pore sizes ranging from ≈1 to 100 μm (Figure 6A–E). The pore size distribution showed a mean pore size of 5.85 ± 9.50 μm in all cases (Figure 6F). Although the different hydrogels had a similar mean pore size, we observed a denser collagen fiber network (Figure 6D,E) in the hydrogels coupled with a higher concentration of GSPs. Furthermore, the number of pores decreased (Figure 6D,E). This fact may be attributed to the fact that GSPs act as cross-linking agents by inducing the formation of hydrogen bonds with collagen [24,31,32].

On the other hand, the percentage of porosity of the hydrogels was also determined, which confirmed that the percentage of porosity decreased with an increasing concentration of GSPs due to cross-linking by them (Figure 6G). Likewise, Figure 6G shows that GSPs at concentrations of 50 and 100 μg·mL^−1^ did not modify the porosity percentage of the hydrogels as there was no significant difference (ns) to the hydrogel without GSPs (0 μg·mL^−1^). However, this was not the case for GSPs at concentrations of 150 and 200 μg·mL^−1^, as there were significant differences (** *p* < 0.0084 and *** *p* < 0.0008) due to the decrease in the porosity percentage. It is worth mentioning that porosity in cellular scaffolds and hydrogels plays an essential role in repairing damaged tissue as it allows cell migration and the diffusion of nutrients and waste [33]. We were even able to confirm that the cardiomyocytes seeded on the surface of the hydrogel migrated to the interior, demonstrating that the hydrogel possessed the appropriate properties for the migration and survival of cardiomyocytes (Video S1).

#### 2.2.4. Gelation Kinetics

**Figure 7:**
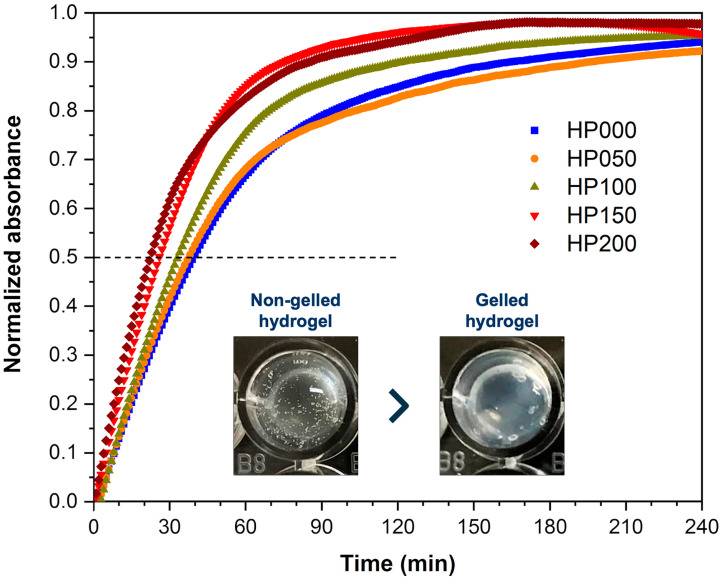
Gelation curves of myocardial ECM hydrogels coupled at different GSPs concentrations (HP000: 0 μg·mL^−1^, HP050: 50 μg·mL^−1^, HP100: 100 μg·mL^−1^, HP150: 150 μg·mL^−1^ and HP200: 200 μg·mL^−1^), *n* = 3.

Turbidity measurements determined the gelation kinetics of the myocardial ECM hydrogels. For this, the absorbance of the hydrogels was measured at a wavelength of 405 nm and a constant temperature of 37 °C for 4 h. The normalized absorbance plotted against time and the calculated parameters of the linear fits (*t*_1/2_ and *S*) are shown in Figure 7 and Table 2, respectively. The addition of GSPs to the hydrogels had a significant effect on the gelation kinetics, as increasing the concentration of GSPs caused a decrease in the gelation time and, thus, an increase in the gelation rate. This fact provides evidence for cross-linking interactions driven by GSPs. The significant change in the macromolecular structure of the hydrogels with the addition of GSPs (Figure 6A–E) and the interference of the passage of light through the hydrogel matrix, once gelation has started, seem to be responsible for the linear increase in absorbance. According to the statistical analysis, the concentrations of 50 and 100 μg·mL^−1^ of GSPs did not modify the gelation time of the hydrogels as there was no significant difference to the hydrogel without GSPs (0 μg·mL^−1^). However, this was not the case for the GSPs at 150 and 200 μg·mL^−1^ concentrations, as there were significant differences (* *p* < 0.0327 and ** *p* < 0.0025) due to the decrease in gelation time.

#### 2.2.5. In Vitro Degradation

**Figure 8:**
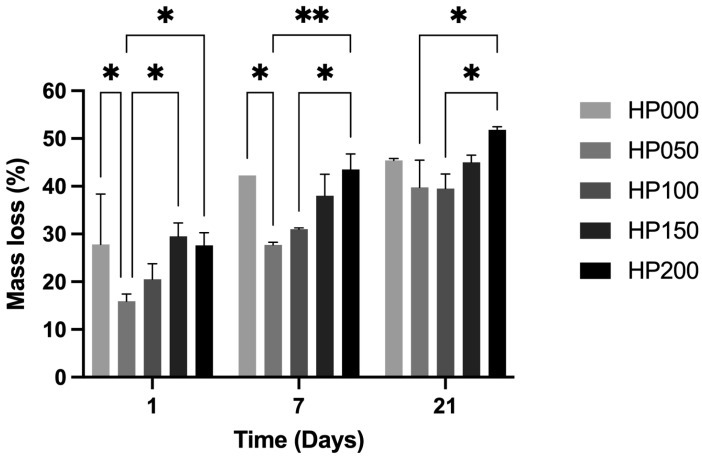
Degradation of myocardial ECM hydrogels coupled at different GSPs concentrations (HP000: 0 μg·mL^−1^, HP050: 50 μg·mL^−1^, HP100: 100 μg·mL^−1^, HP150: 150 μg·mL^−1^ and HP200: 200 μg·mL^−1^) at different incubation times with PBS (1, 7, and 21 days). The increase in the percentage of mass loss indicates a more significant degradation. * *p* < 0.0334 and ** *p* < 0.0060 indicate a significant difference between the treatments, *n* = 3.

The effect of GSPs on the degradation of myocardial ECM hydrogels was assessed by hydrolysis with PBS. Figure 8 shows that hydrogels coupled with a low concentration of GSPs (50 and 100 μg·mL^−1^) showed lower degradation than the other concentrations tested (0, 150, and 200 μg·mL^−1^). Other studies have already indicated the behavior of collagen degradation when it is cross-linked with GSPs, where it is proven that very low doses of GSPs (from 0.5% to 6.5%) are sufficient to cross-link collagen, while higher doses (for example 20%) decrease the degradation rate [34,35]. In addition to the above, it has been emphasized that, given the antioxidant capacity of GSPs, it is likely that when they are used in higher concentrations, the cross-linking rates decrease between collagen molecules, especially due to the antioxidant properties of GPSs, which could interfere with the polymerization of free radicals of the adhesive monomers of collagen [36]. The degradation rate can also be affected by the application time of GSPs, which is generally from one minute to one hour [34,37,38]. In the present manuscript, the GSPs are part of the hydrogel; therefore, free GSPs with antioxidant properties may promote less cross-linking between the peptides, which would be involved with the greater mass loss observed. The constructed hydrogels could be used as a treatment for cardiac injuries, such as myocardial infarction. Thereby, slow degradation of the hydrogel in the infarct or border zone could be beneficial to ensure that the scaffold provides the extracellular medium for cell survival and, on the other hand, degrades at a rate that facilitates the repair of damaged tissue [8]. Although some studies indicate that the in vitro degradation time of hydrogels does not correlate with the in vivo degradation time due to multiple factors [39,40], the results we obtained suggest that coupling GSPs at a low concentration (50 and 100 μg·mL^−1^) will likely decrease the degradation of hydrogels in vivo.

### 2.3. Biological Evaluation of Hydrogels

#### 2.3.1. Cell Viability

**Figure 9:**
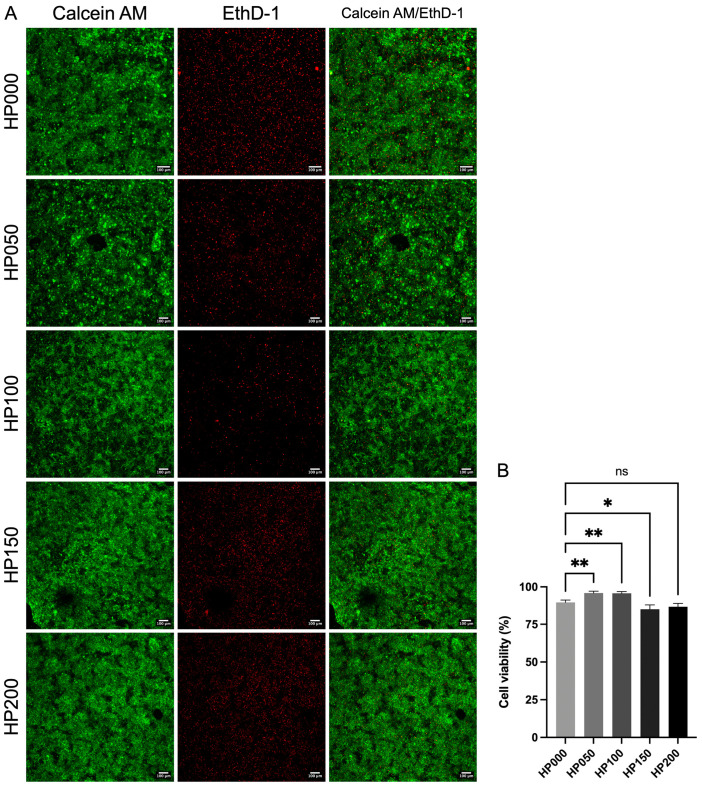
Viability of chicken embryo cardiomyocytes cultured on myocardial ECM hydrogels coupled at different GSPs concentrations (HP000: 0 μg mL^−1^, HP050: 50 μg mL^−1^, HP100: 100 μg mL^−1^, HP150: 150 μg mL^−1^ and HP200: 200 μg mL^−1^). (**A**) Representative photomicrographs of cell viability in hydrogels coupled at different GSPs concentrations, *n* = 4. Live cardiomyocytes are labeled with calcein AM (first column), dead cardiomyocytes are labeled with ethidium homodimer-1 (second column), and the overlay of both fluorescent dyes is shown in the third column. (**B**) Percentage of cell viability in hydrogels coupled at different GSPs concentrations. ns indicates no significant difference between the treatments. * *p* < 0.0315 and ** *p* < 0.0039 indicate a significant difference between the treatments, *n* = 4.

The viability of chicken embryo cardiomyocytes cultured on myocardial ECM hydrogels was assessed by fluorescent calcein AM/ethidium homodimer-1 staining: calcein AM evidence live cells by the activity of the ubiquitous intracellular esterase that converts non-fluorescent calcein AM to green-fluorescent calcein. Ethidium homodimer-1 enters cells with damaged membranes (dead cells) and produces a red fluorescence upon binding to nucleic acids [41]. Representative photomicrographs of calcein AM/ethidium homodimer-1 show that cardiomyocytes cultured on the hydrogels exhibited relatively high viability (Figure 9A). We observed that the hydrogels coupled with a low concentration of GSPs (50 and 100 μg·mL^−1^) showed a lower number of dead cells (marked in red) compared to the other concentrations evaluated (0, 150, and 200 μg·mL^−1^). The percentage of cell viability that was calculated from the photomicrographs showed that (Figure 9B) the percentage of cell viability increased in the hydrogels coupled with GSPs at the concentrations of 50 and 100 μg·mL^−1^ (95.9 ± 1.2 and 95.7 ± 1.1%, respectively) compared to the hydrogel without GSPs (89.7 ± 1.6%). The difference between them was significant (*p* < 0.0039). The cell viability percentage of the hydrogels coupled with GSPs at 150 and 200 μg·mL^−1^ (85.0 ± 2.9 and 86.7 ± 2.3%, respectively) decreased similarly to the hydrogel without GSPs. These results were consistent with the study of Zhai et al. [42], which demonstrated that GSPs in a concentration range of 31.3 to 125.0 μg·mL^−1^ induce the proliferation of interstitial cells of the bovine heart valve, while GSPs at a higher concentration (>125.0 μg·mL^−1^) had an inhibitory effect on cell proliferation.

#### 2.3.2. Cell Functionality

**Figure 10:**
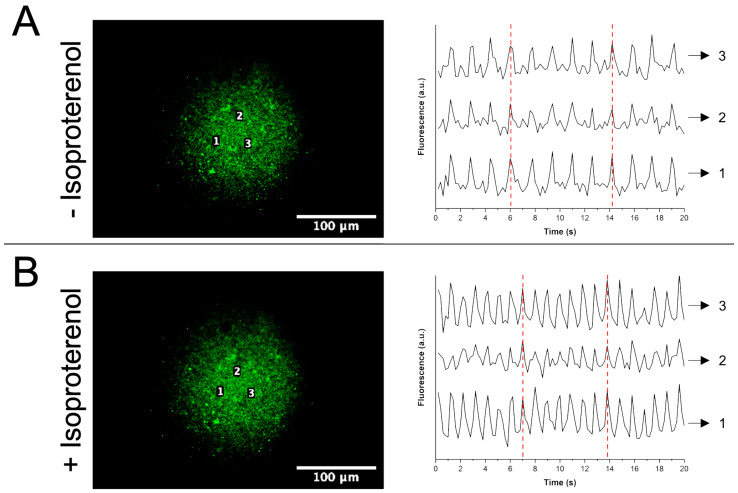
Contractile activity patterns of chicken embryo cardiomyocytes cultured on myocardial ECM hydrogel coupled with 100 μg·mL^−1^ GSPs at 24 h of culture: (**A**) without isoproterenol and (**B**) with isoproterenol, *n* = 3. The numbers in the photomicrographs (1, 2, and 3) represent the sites in the cell cultures where the contractile activity patterns were obtained. The red dashed lines in the contractile activity patterns indicate how the wave crests coincide between the different numberings.

Since the viability of chicken embryo cardiomyocytes cultured on myocardial ECM hydrogels coupled with 50 and 100 μg·mL^−1^ GSPs was similar to, but higher than shown on hydrogels coupled with 150 and 200 μg·mL^−1^ GSPs (Figure 9B), the contractile activity of cardiomyocytes was analyzed on the hydrogel coupled with 100 μg·mL^−1^ GSPs using the fluorescent indicator calcium green-1. Figure 10A shows that cardiomyocytes maintained contractile activity at 24 h of culture. Furthermore, it is worth mentioning that cardiomyocytes showed synchronous contractility since the wave crests coincide between the different contractile activity patterns analyzed. When the cultures were treated with isoproterenol, it was observed that the contractility frequency of cardiomyocytes increased (Figure 10B). The contractility frequency of cardiomyocytes without isoproterenol was ≈0.6 cycles per second (Figure 10A), while with isoproterenol it was ≈1.0 cycle per second (Figure 10B). This experiment demonstrated that myocardial ECM hydrogel coupled with 100 μg·mL^−1^ GSPs does not inhibit cardiomyocyte functionality in vitro.

## 3. Conclusions

The protocol to decellularize porcine myocardium effectively removed cells, as evidenced by histological evaluation and DNA quantification, which indicated that the myocardial ECM hydrogel would not induce an adverse immune response. Furthermore, the myocardial decellularization process retained ECM components such as collagen and proteoglycans, allowing cell adhesion, viability, and proliferation. On the other hand, the ECM solution coupled with GSPs can self-assemble into a hydrogel at a temperature of 37 °C. Vibrational spectroscopy demonstrated that GSPs act as cross-linking agents by inducing the formation of hydrogen bonds with collagen, which did not significantly alter the triple helix conformation of this protein. Although the antioxidant capacity of the hydrogels coupled with GSPs decreased due to cross-linking interactions, it was very high compared to the hydrogel without GSPs. Furthermore, the incorporation of GSPs significantly affected the porosity and gelation time of the hydrogels since the increase in the concentration of GSPs induced a decrease in the porosity and gelation time. Furthermore, the coupling of GSPs at low concentrations positively affected the in vitro degradation and cell viability of the hydrogels. Likewise, the functional activity of cardiomyocytes was not inhibited when they were cultured on the hydrogel coupled with 100 μg·mL^−1^ GSPs. Thus, the GSPs concentrations that we propose for future studies in cardiac tissue engineering are the concentrations of 50 and 100 μg·mL^−1^ since the hydrogels at these concentrations not only exhibited a high antioxidant capacity, a shorter gelation time, and a similar porosity to the hydrogel without GSPs. In addition, the in vitro degradation and cell viability are not compromised. Overall, the results of this work demonstrate that the incorporation of GSPs into the thermosensitive myocardial ECM hydrogel represents a promising alternative for its potential application in myocardial regeneration after a heart attack.

## 4. Materials and Methods

### 4.1. Obtaining Porcine Myocardial ECM

For obtaining ECM, porcine ventricular tissue was decellularized according to some modifications of previously published protocols [9,21,43,44]. Porcine hearts were obtained immediately after performing the anesthesia and euthanasia procedure in healthy 6-month-old Yorkshire pigs weighing approximately 100 kg. Anesthesia consisted of an intramuscular injection of ketamine (25 mg·kg^−1^)/xylazine (2 mg·kg^−1^), and for euthanasia, an overdose of sodium pentobarbital (90 mg·kg^−1^) was administered in the marginal vein of the ear. The left ventricle was isolated from a porcine heart, and the superficial fat, valve, chordae tendineae, and papillary muscles were removed, leaving only the myocardium. The tissue was cut into small regular cubes and placed in distilled water under shaking at 125 rpm for 45 min to remove excess blood. Subsequently, the tissue was shaken in a 1% (*m*/*v*) sodium dodecyl sulfate (SDS) solution in phosphate-buffered saline (PBS) at 125 rpm for 4-5 days with a solution change every 24 h until the tissue turned completely white. The tissue was then rinsed with distilled water to remove SDS and incubated in a DNase I solution (from bovine pancreas, >2000 U·mg^−1^, 11284932001, Roche Diagnostics Deutschland GmbH, Mannheim, Germany) at 37 °C under shaking (125 rpm) for 24 h. The DNase I solution consisted of 0.1 mg·mL^−1^ DNase I in 6 mM magnesium chloride hexahydrate (MgCl_2_·6H_2_O), 1 mM calcium chloride (CaCl_2_), and 10 mM sodium chloride (NaCl), at a pH of 7.4. After incubation, the tissue was rinsed with distilled water and shaken in 1% SDS solution at 125 rpm for 24 h. Finally, the tissue was placed in 0.001% (*v*/*v*) Triton X-100 solution in PBS under shaking at 125 rpm for 1 h and rinsed with distilled water.

### 4.2. Evaluation of Myocardial ECM

To verify the efficacy of the decellularization process and to check that the myocardial ECM contained components such as collagen and proteoglycans, the native and decellularized tissues were processed using conventional histological techniques and stained with H&E (Abcam Limited, Cambridge, UK), DAPI (Thermo Fisher Scientific Inc., Waltham, MA, USA), Russell-Movat pentachrome (Abcam Limited, Cambridge, UK), and Alcian blue (Abcam Limited, Cambridge, UK).

Furthermore, the presence of the α-Gal epitope in the native and decellularized tissues was examined by indirect immunohistochemistry. The α-Gal molecule is expressed in pigs and is involved in the immunological rejection of xenotransplants [17,18]. For this, the samples were placed in a 0.6 M citrate buffer (pH 6) at 120 °C for 3 min to perform antigen retrieval, incubated in 3% hydrogen peroxide for 10 min to block endogenous peroxidase and washed with 5% albumin/1% Tween 20 at 37 °C for 1 h to block nonspecific sites and permeabilize the membranes. Subsequently, they were incubated with a mouse IgM primary antibody against Galα1-3Galβ1-4GlcNAc-R (1:5, Enzo Life Sciences, Inc., Farmingdale, NY, USA) at 4 °C for 18 h and then with a biotinylated rabbit secondary antibody against mouse IgM (1:1000) at 37 °C for 1 h. Finally, they were incubated with the streptavidin/peroxidase complex (HRP, 1:1200) at 37 °C for 45 min, then incubated with the chromogen diaminobenzidine (DAB) 1X for 5 min, and the samples were counterstained with hematoxylin for 3 min. All stained tissues were observed using an ECLIPSE 80i microscope (Nikon Instruments Inc., Melville, NY, USA).

To quantitatively assess the degree of decellularization, DNA was isolated from native and decellularized tissues using the phenol/chloroform/isoamyl alcohol method and quantified (*n* = 3) using a NanoDrop™ 2000 spectrophotometer (Thermo Fisher Scientific Inc., Waltham, MA, USA).

### 4.3. Fabrication of Myocardial ECM Hydrogel

The hydrogel was prepared according to some modifications of previously published protocols [9,43,44]. Myocardial ECM was lyophilized at a vacuum pressure of 0.037 hPa and a temperature of −109 °C for 18 h using a CoolSafe™ device (LaboGene ApS, Vassingerød, Denmark). The lyophilized ECM was then pulverized using liquid nitrogen and strained through a #40 fine mesh metal strainer. Then, ECM powder (10 mg·mL^−1^) was placed in a solution of pepsin (from porcine gastric mucosa, ≥250 U·mg^−1^, P7000, Sigma-Aldrich Inc., St. Louis, MO, USA) 1 mg·mL^−1^ in 0.01 M HCl and digested at 125 rpm and 25 °C for 12 h to solubilize ECM. Then, a GSPs solution (Tianjin Jianfeng Natural Product R&D Co., Ltd., Teda, Tianjin, China) of 1 mg·mL^−1^ was added to the ECM solution in appropriate volumes so that GSPs were at concentrations of 0, 50, 100, 150 and 200 μg·mL^−1^. The pH was then adjusted to 7.4 with NaOH solution to bring the ECM in liquid form to a physiological pH and inactivate pepsin. Once the ECM solution was neutralized, the total volume of the solution was calculated, and 1/9 of this volume of 10X PBS was added to bring the solution to a 1X PBS salt concentration and mimic physiological conditions. Finally, the ECM solution was diluted to 6 mg·mL^−1^ with 1X PBS and incubated at 37 °C overnight to self-assemble into a hydrogel. According to the certificate of analysis of the GSPs, they come from the grape seed extract of the species *Vitis vinifera* L., which contains at least 95% GSPs. (+)-catechin and (-)-epicatechin are the GSPs found in significant quantities in the monomeric or oligomeric form of the extract. However, the GSPs that are present in the majority are the oligomeric ones, with at least 60%.

### 4.4. Physicochemical Evaluation of Hydrogels

#### 4.4.1. Infrared Spectroscopy

The interaction of myocardial ECM and GSPs was analyzed by Fourier transform infrared spectroscopy (FTIR) using a Spectrum 400 spectrometer (PerkinElmer Inc., Waltham, MA, USA) connected to an attenuated total reflectance (ATR) diamond cell. Each spectrum had an average of 32 scans with a resolution of 4 cm^−1^.

#### 4.4.2. Antioxidant Capacity

The antioxidant capacity of myocardial ECM hydrogels was determined using a modified 2,2-diphenyl-1-picrylhydrazyl (DPPH) assay [45]. In microcentrifuge tubes, 250 μL of the ECM hydrogels coupled at different GSPs concentrations (0, 50, 100, 150 and 200 μg·mL^−1^) were added, and then 750 μL of the 100 μM DPPH• solution was added. Subsequently, the tubes were shaken and incubated in the dark at 37 °C for 30 min. After incubation, the tubes were centrifuged at 1 × 10^4^ rpm at 4 °C for 5 min, and 200 μL of the supernatant was placed in a 96-well plate (*n* = 3). Finally, absorbance was measured at a wavelength of 540 nm using a Multiskan™ Sky plate reader (Thermo Fisher Scientific Inc., Waltham, MA, USA). A 75 μM DPPH• solution was used as a control. Antioxidant capacity was calculated using the following equation:

(1) $$DPPH\;reduction\;\left(\%\right)=\frac{A_{Ctrl}-A_{Spl}}{A_{Ctrl}}\cdot 100$$

where *A_Ctrl_* is the absorbance of the control, and *A_Spl_* is the absorbance of the sample.

#### 4.4.3. Scanning Electron Microscopy

The morphology of myocardial ECM hydrogels was observed by scanning electron microscopy (SEM, JSM-7800F, JEOL Ltd., Akishima, Tokio, Japan) in secondary electron mode with an accelerating voltage of 2 kV under a high vacuum. Before observing the ECM hydrogels coupled at different GSPs concentrations, they were frozen by immersion in liquid nitrogen and dehydrated by lyophilization at a vacuum pressure of 0.038 hPa and a temperature of −109 °C for 12 h to maintain the microstructure of the hydrated state. Finally, the dehydrated hydrogels were coated with gold by plasma-assisted physical vapor deposition (PAPVD, Hummer VI-A, Anatech Ltd., Alexandria, VA, USA). The diameter and area of the pores were measured using the image processing and analysis software ImageJ 1.54m (National Institutes of Health, USA). The percentage of porosity was calculated using the following equation:

(2) $$Porosity\;\left(\%\right)=\frac{a_{Pore}}{a_{Total}}\cdot 100$$

where *a_Pore_* is the pore area, and *a_Total_* is the total area.

#### 4.4.4. Gelation Kinetics

The gelation kinetics of myocardial ECM hydrogels was determined by turbidity measurements [46]. ECM hydrogels coupled at different GSPs concentrations were prepared as previously described, and 100 μL of the hydrogel solution on a 96-well plate (*n* = 3) was placed. The absorbance was measured at a wavelength of 405 nm every minute for 4 h to 37 °C using a Cytation™ 5 plate reader (BioTek^®^ Instruments Inc., Winooski, VT, USA). The absorbance values were normalized using equation 3 and are represented according to time. From the standardized graph, a linear adjustment was applied to the linear region of the graph to calculate the average gelation time (*t*_1/2_) and the slope or speed of the gelation (*S*). The average gelation time was defined as the time when the hydrogel reached 50% of the maximum measure.

(3) $$Normalized\;absorbance=\frac{A_{x}-A_{min}}{A_{max}-A_{min}}$$

where *A_x_* is the experimental absorbance, *A_min_* is the minimum absorbance, and *A_max_* is the maximum absorbance.

#### 4.4.5. In Vitro Degradation

The degradation of myocardial ECM hydrogels was assessed by hydrolysis with PBS. ECM hydrogels coupled at different GSPs concentrations were allowed to gel overnight in microcentrifuge tubes with volumes of 300 μL (*n* = 3). They were placed in an equal volume of PBS and subsequently incubated at 37 °C at different contact times (0, 1, 7, and 21 d). At the end of each contact time, the PBS was removed, and the hydrogels were dehydrated by lyophilization at a vacuum pressure of 0.037 hPa and a temperature of −109 °C for 9 h. Finally, the mass of the dehydrated hydrogels was measured to calculate the percentage of mass loss using the following equation:

(4) $$Mass\;loss\;\left(\%\right)=\frac{M_{0}-M_{t}}{M_{t}}\cdot 100$$

where *M*_0_ is the mass of the dehydrated hydrogel in contact with PBS at time 0 d, and *M_t_* is the mass of the dehydrated hydrogel that was in contact with PBS at time 1, 7, or 21 d.

### 4.5. Biological Evaluation of Hydrogels

#### 4.5.1. Cell Viability

The viability of cells cultured on myocardial ECM hydrogels was assessed using ventricular cardiomyocytes from White Leghorn chicken embryos at 7 days of in ovo development [47,48]. We obtained fertilized and specific pathogen-free eggs from a local supplier (ALPES^®^, Tehuacán, Puebla, Mexico). The culture medium to maintain cardiomyocytes contained M199 (Gibco™, Thermo Fisher Scientific Inc., Waltham, MA, USA), 17 mM HEPES, 10% horse serum (HS), 5% fetal bovine serum (FBS), and 50 μg·mL^−1^ gentamicin.

The viability of cardiomyocytes cultured on ECM hydrogels was assessed by calcein AM/ethidium homodimer-1 fluorescent staining (Invitrogen™, Thermo Fisher Scientific Inc., Waltham, MA, USA). Hydrogels coupled at different GSPs concentrations were allowed to gel overnight in 1 cm diameter molds with volumes of 200 μL (*n* = 4) and then rinsed with Hanks balanced salt solution (HBSS) with 9.9 mM HEPES at a pH of 7.2. Subsequently, cardiomyocytes were seeded on the hydrogels (2.25 × 10^6^ cells/hydrogel) in 200 μL of culture medium and incubated at 37 °C, in a 5% CO_2_ atmosphere, and with 100% humidity. After 24 h of incubation, the culture medium was replaced with 200 μL of the staining solution (calcein AM 0.5 μL·mL^−1^/ethidium homodimer-1 2 μL·mL^−1^) and incubated at 37 °C for 30 min. Finally, the staining solution was replaced with 200 μL of HBSS with HEPES and observed under a TCS SP8 confocal microscope (Leica Microsystems GmbH, Wetzlar, Germany) at the excitation/emission wavelengths 494/517 nm (calcein AM) and 528/617 nm (ethidium homodimer-1). The area of the calcein AM and ethidium homodimer-1 fluorescent labels was measured using the image processing and analysis software Fiji 2.16.0. The viability percentage was calculated using the following equation:

(5) $$Cell\;viability\;\left(\%\right)=\frac{a_{Calcein\;AM}}{a_{Calcein\;AM}+a_{EthD-1}}\cdot 100$$

where *a_Calcein AM_* is the area of the calcein AM fluorescent label and *a*_*EthD*−1_ is the area of the ethidium homodimer-1 fluorescent label.

#### 4.5.2. Cell Functionality

Once cell viability on myocardial ECM hydrogels was assessed, contractile activity of cardiomyocytes was studied on the hydrogel coupled with 100 μg·mL^−1^ GSPs using the fluorescent indicator calcium green-1 (Invitrogen™, Thermo Fisher Scientific Inc., Waltham, MA, USA), which increases fluorescence intensity upon binding to intracellular Ca^+2^. The protocol followed was similar to that for cell viability (Section 4.5.1). On the cultures, 200 μL of the indicator solution (10 μg·mL^−1^ calcium green-1/20 μL·mL^−1^ DMSO /0.4% pluronic acid) were placed and incubated at 37 °C. After 45 min of incubation, the indicator solution was replaced by HBSS and observed using a MZ75 microscope (Leica Microsystems GmbH, Wetzlar, Germany) at excitation/emission wavelengths of 506/531 nm. A total of 300 images were taken for 1 min at an exposure time of 75 ms for further analysis using ImageJ 1.54m software (National Institutes of Health, USA). Some cultures were treated with 1 μM isoproterenol in order to analyze the functional response of cardiomyocytes cultured on the hydrogel coupled with 100 μg·mL^−1^ GSPs. The contractility frequency of cardiomyocytes was calculated using the following equation:

(6) $$f=\frac{1}{T}$$

where *f* is the frequency in cycles per second and *T* is the time to complete one cycle in seconds.

### 4.6. Statistical Analysis

Data were expressed as mean ± SD and statistically compared by one-way and two-way analysis of variance (ANOVA) with a Tukey post-hoc test using Prism 10.4.1 statistical software (GraphPad Software LLC., San Diego, CA, USA), considering *p* < 0.05 as statistically significant.

## Figures and Tables

**Table 1:** Ratios of absorption intensities of the Amide III band and the band at 1450 cm^−1^ for the myocardial ECM hydrogels coupled at different GSPs concentrations.

Hydrogel	GSPs (μg·mL^−1^)	A_III_/A_1450_
HP000	0	1.02
HP050	50	1.06
HP100	100	1.07
HP150	150	1.06
HP200	200	1.06

**Table 2:** Rate (*S*) and mean gelation time (*t*_1/2_) of myocardial ECM hydrogels coupled at different GPSs concentrations.

Hydrogel	GSPs (μg·mL^−1^)	*S*	*t*_1/2_ (min)
HP000	0	0.0158 ± 0.0016	33.3 ± 3.0
HP050	50	0.0189 ± 0.0002	28.2 ± 0.5
HP100	100	0.0202 ± 0.0031	26.9 ± 3.3
HP150	150	0.0214 ± 0.0031	24.6 ± 3.2 *
HP200	200	0.0237 ± 0.0050	18.1 ± 1.6 **

* *p* < 0.0327 and ** *p* < 0.0025 indicate that there is a significant difference with respect to the hydrogel without GSPs, *n* = 3.

## Data Availability

The original contributions presented in this study are included in the article/Supplementary Material. Further inquiries can be directed to the corresponding author.

## Supplementary Materials

The following supporting information can be downloaded at: https://www.mdpi.com/article/10.3390/gels11010053/s1, Video S1: Chicken embryo cardiomyocytes within thermosensitive porcine myocardial ECM hydrogel coupled with 50 μg·mL^−1^ GSPs stained with calcein AM/ethidium homodimer-1.
